# Nursing Students Visiting People with Dementia Online during COVID-19: A Qualitative Study

**DOI:** 10.1093/geroni/igab046.3324

**Published:** 2021-12-17

**Authors:** Michelle Kimzey, Jodi Patterson, Beth Mastel-Smith

**Affiliations:** 1 Harris College of Nursing & Health Sciences (Texas Christian University), Fort Worth, Texas, United States; 2 TCU, Fort Worth, Texas, United States; 3 University of Texas at Tyler, Tyler, Texas, United States

## Abstract

The coronavirus disease 2019 (COVID-19) crisis has impacted the daily routines of students, people living with dementia, and their care partners. Social distancing results in fewer interpersonal interactions and enjoyable activities which makes life more challenging for those living with dementia. The purposes of this multiple case study were to understand how nursing students, people with dementia, and care partners (a) describe online visits between nursing students and people with dementia during stay-at-home directives in response to COVID-19 and (b) the perceived visit benefits. Nursing students participated in online visits to socially engage with their mentor (person living with dementia). During the visits it was anticipated that care partners would enjoy a brief respite. After 12 visits, investigators completed one-to-one online interviews with students (n = 10), care partners (n = 8) and mentors (n = 8). All cases reported a positive experience, perceptions of the conversations, improved social connection and meaning and purpose, mentor’s enhanced cognition and planned future connections. Relationships were formed between students, people with dementia, and care partners during online visits, an activity that might be implemented outside of a crisis to prevent social isolation across generations. Future efforts to engage people with dementia in residential facilities should be formally integrated into the care plan and staff dedicated to help with technology assigned.

